# Robust increase in glucagon secretion after oral protein intake, but not after glucose or lipid intake in Japanese people without diabetes

**DOI:** 10.1111/jdi.14053

**Published:** 2023-07-21

**Authors:** Raishi Ichikawa, Koji Takano, Kazumi Fujimoto, Masaki Kobayashi, Tadahiro Kitamura, Masayoshi Shichiri, Takeshi Miyatsuka

**Affiliations:** ^1^ Department of Diabetes, Endocrinology, Diabetes and Metabolism Kitasato University, School of Medicine Sagamihara Japan; ^2^ Metabolic Signal Research Center, Institute for Molecular and Cellular Regulation Gunma University Maebashi Japan

**Keywords:** Glucagon, Lipid load, Protein load

## Abstract

Few studies in Asian populations have analyzed how glucagon secretion is affected by ingested glucose, proteins or lipids, individually. To investigate the fluctuations of glucagon secretion after the intake of each of these nutrients, 10 healthy volunteers underwent oral loading tests using each of glucose, proteins and lipids, and blood levels of glucose, insulin and glucagon were measured every 30 min for 120 min. Whereas glucagon secretion was suppressed and minimally affected by oral glucose intake and lipid intake, respectively, oral protein intake robustly increased glucagon secretion, as well as insulin secretion. Further studies are needed to elucidate the mechanism by which protein loading increases glucagon secretion.

## Graphical abstract

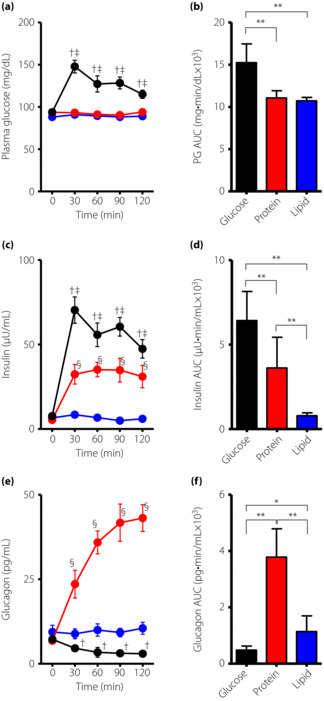

## INTRODUCTION

Several studies have reported the fluctuation of plasma glucagon levels after food intake in both people with and without diabetes, showing no consistent patterns of glucagon profiles^1^, ^2^. This variation in glucagon secretion in response to food intake might be attributed to the different compositions of carbohydrates, proteins and lipids in various foods. Although there have been several reports examining glucagon responses to macronutrient intake^3^, ^4^, ^5^, ^6^, ^7^, ^8^, there have been arguments regarding the accuracy of glucagon measurement, as glucagon secretion was evaluated by conventional radioimmunoassay in most studies^3^, ^4^, ^5^. Furthermore, very few reports to date have investigated glucagon responsiveness to glucose, protein and lipid in a single study^4^, ^6^, ^8^. To properly investigate the fluctuations of glucagon secretion after the intake of each nutrient, plasma glucagon profiles were measured using a sandwich‐type enzyme‐linked immunosorbent assay method after the intake of carbohydrates, proteins or lipids, separately, in 10 Japanese people without diabetes.

## METHODS

Participants were 10 healthy Japanese volunteers (four men, six women), aged 27.9 ± 3.7 years (range 20–40 years) with a body mass index of 20.4 ± 2.3 (17.2–24.8). Exclusion criteria are as follows: pregnant or possibly pregnant, lactating mothers, having acute illness, taking medications regularly, past history of gastrointestinal operation or malignancy, allergic to whey protein and ≥5% weight gain or loss within the past 1 month. Participants who showed abnormal glucose tolerance in the 75‐g oral glucose tolerance test (OGTT) were also excluded.

All participants underwent oral loading tests using each of glucose, proteins and lipids. Each test was carried out after at least a 1‐week interval. The participants were instructed to fast overnight for a minimum of 10 h before the test. Blood samples were collected before, and 30, 60, 90 and 120 min after the intake of each nutrient, and plasma glucose, insulin and glucagon were measured as previously described^9^. The OGTT was carried out with TRELAN‐G75 (AY Pharmaceuticals Co., Ltd., Tokyo, Japan). The protein loading test was carried out with 81 g of protein powder (SAVAS PRO CLEAR PROTEIN WHEY 100; Meiji Co., Ltd., Tokyo, Japan), containing 97% whey protein, and the lipid loading test was carried out with 34 g of pure virgin olive oil (Inoue Seikoen, Kagawa, Japan), to match the energy intake of 75 g of glucose in the OGTT. Biochemical analyses were carried out using the same methods as previously described^10^. Plasma glucagon was measured using a sandwich‐type enzyme‐linked immunosorbent assay kit (Mercodia, Uppsala, Sweden).

All laboratory measurements are presented as the mean ± standard error of the mean. The area under the curve of plasma glucose, insulin and glucagon was calculated by using the trapezoidal method^11^. Univariate tests for differences in values among the three groups were carried out using the one‐way anova. If a significant difference was observed in anova, post‐hoc comparisons were carried out using Tukey's honestly significant difference test. Statistical analyses were carried out using of JMP software version 10 (SAS Institute Inc., Tokyo, Japan), and a *P*‐value of <0.05 was considered to show a statistically significant difference between groups.

## RESULTS

**Figure 1:**
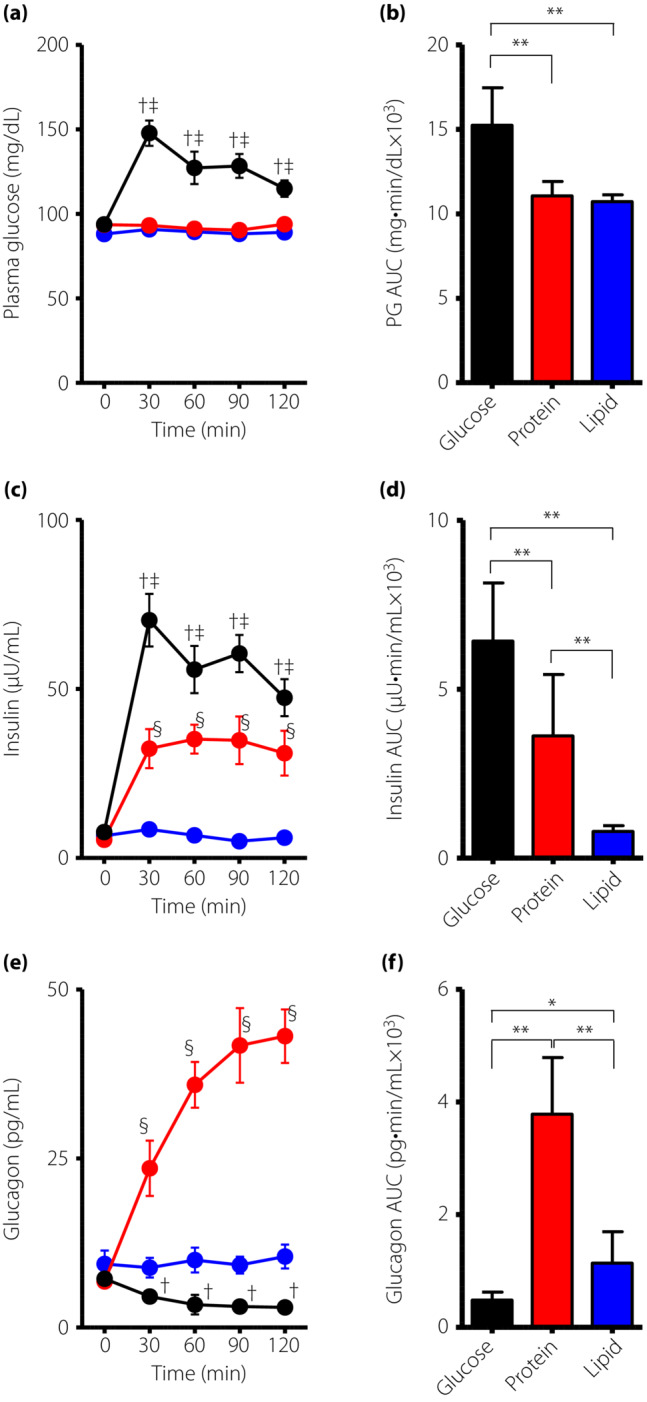
Dynamic changes in plasma glucose (PG) levels and endocrine parameters during the 75‐g oral glucose tolerance test, and protein and lipid loading tests in young healthy people without diabetes. Levels of (a, b) PG, (c, d) insulin and (e, f) glucagon were measured during the 75‐g oral glucose tolerance test (black), protein loading test (red) and lipid loading test (blue). Data are presented as the mean ± standard error of the mean. ^†^ *P* < 0.05 versus protein loading test, ^‡^ *P* < 0.05 versus lipid loading test, ^§^ *P* < 0.05 protein loading test versus lipid loading test. *P < 0.05, **P < 0.01. AUC, area under the curve.

During the OGTT, plasma glucose levels were significantly increased after a glucose load, whereas they were almost unchanged after both protein and lipid loads (Figure 1a,b). Plasma insulin levels were increased after both glucose and protein loads, but no increase in insulin level was observed after a lipid load (Figure 1c,d). Insulin levels during the OGTT were significantly higher than those during protein and lipid loading tests. Insulin levels after a protein load were significantly higher than after a lipid load. Whereas plasma glucagon levels were significantly decreased after a glucose load, they were robustly elevated after protein load (Figure 1e,f). Lipid intake did not affect glucagon secretion.

## DISCUSSION

It has been shown that postprandial glucagon secretion as well as insulin secretion are impaired in people with diabetes^6^, ^10^, ^12^; however, few studies to date have analyzed how much each nutrient contributes to plasma glucagon secretion in Asian populations. In the present study, we measured plasma glucagon profiles after three consecutive loading tests for three major nutrients, namely, carbohydrates, proteins and lipids, in 10 Japanese participants without diabetes, and clearly showed that only protein intake robustly increases glucagon secretion, whereas both lipid intake and carbohydrate intake have minimal effects on glucagon secretion. These findings in Japanese people are consistent with the results of the previous study on white people^6^.

Previous *ex vivo* experiments in rats using perfused pancreata showed that glucagon secretion was suppressed by glucose and was promoted by amino acids^13^, ^14^, whereas free fatty acids tended to suppress glucagon secretion^15^. It has been proposed that glucagon and insulin secretion are regulated by intraportal glucose levels, and glucose sensing signals from intraportal glucose sensors are projected to the hypothalamic nuclei involved in the regulation of glucagon and insulin secretion by an afferent pathway^16^, ^17^, ^18^. A clinical study showed that both 30 and 70 g of whey protein stimulated glucagon secretion in healthy men, without increasing blood glucose levels^19^, which is consistent with the present data.

Our experiments showed that a protein load promotes both insulin and glucagon secretion (Figure 1c–f), implying that glucagon secretion is increased to maintain blood glucose levels in response to increased insulin through glycogenolysis and gluconeogenesis. Further time‐resolved studies are required to clarify the physiological interaction between insulin and glucagon secretion after protein intake. In contrast, it remains unclear as to which amino acid(s) is responsible for the robust increase in glucagon secretion after protein intake. Oral loading tests with different types of proteins/amino acids are expected to provide further insight into glucagon secretion profiles after protein intake.

In conclusion, the present study showed that glucagon secretion is suppressed by carbohydrate intake, and increased by protein intake, whereas oral lipid intake had little effect to glucagon secretion in young healthy people. Whereas protein intake robustly stimulated both insulin and glucagon secretion, plasma glucose levels were maintained at a constant level. Further investigations are needed to elucidate the underlying mechanisms by which protein intake stimulates the dynamic changes in insulin and glucagon secretions, while maintaining plasma glucose levels.

## DISCLOSURE

The authors declare no conflict of interest.

Approval of the research protocol: This study protocol was approved by the Kitasato University Medical Ethics Organization (KMEO), Institutional Review Board for Clinical Research and Treatment (study approval no.: B16‐213).

Informed consent: Written informed consent was obtained from all participants.

Registry and the registration no. of the study/trial: The study was registered at University Hospital Medical Information Network (UMIN) Center (approval no.: 000026502) on 10 March 2017.

Animal studies: N/A.
